# Transcriptome Analysis of Bovine Ovarian Follicles at Predeviation and Onset of Deviation Stages of a Follicular Wave

**DOI:** 10.1155/2016/3472748

**Published:** 2016-03-21

**Authors:** Pengfei Li, Jinzhu Meng, Wenzhong Liu, George W. Smith, Jianbo Yao, Lihua Lyu

**Affiliations:** ^1^College of Life Science, Shanxi Agricultural University, Taigu, Shanxi 030801, China; ^2^College of Animal Science and Technology, Shanxi Agricultural University, Taigu, Shanxi 030801, China; ^3^Laboratory of Mammalian Reproductive Biology and Genomics, Departments of Animal Science and Physiology, Michigan State University, East Lansing, MI 48824, USA; ^4^Division of Animal and Nutritional Sciences, West Virginia University, Morgantown, WV 26506, USA

## Abstract

For two libraries (PDF1 and ODF1) using Illumina sequencing 44,082,301 and 43,708,132 clean reads were obtained, respectively. After being mapped to the bovine RefSeq database, 15,533 genes were identified to be expressed in both types of follicles (cut-off RPKM > 0.5), of which 719 were highly expressed in bovine follicles (cut-off RPKM > 100). Furthermore, 83 genes were identified as being differentially expressed in ODF1 versus PDF1, where 42 genes were upregulated and 41 genes were downregulated. KEGG pathway analysis revealed two upregulated genes in ODF1 versus PDF1, CYP11A1, and CYP19A1, which are important genes in the steroid hormone biosynthesis pathway. This study represents the first investigation of transcriptome of bovine follicles at predeviation and onset of deviation stages and provides a foundation for future investigation of the regulatory mechanisms involved in follicular development in cattle.

## 1. Introduction

The ovarian follicle is an essential component of the reproductive process. It plays an important role in controlling the estrous cycle, determining estrous behaviour, ensuring oocyte competency and subsequent embryo survival rate, and determining both postovulation corpus luteum function and progesterone synthesis [1]. In a number of species, follicular growth is characterized by a wave-like pattern, with two or three waves occurring during the normal course of estrous cycles in cattle [2]. During each wave of follicular development, a cohort of antral follicles are induced to begin accelerated growth [3]. After a period of concurrent growth, a species specific number of follicles will then be selected to become dominant, while the remaining follicles will be lost through a process known as atresia. Diameter deviation is defined as the divergence in growth rates between the two largest follicles in a follicular wave [3]. The onset of diameter deviation occurs when the largest follicle reaches 8.5 mm in dairy cattle and marks initiation of divergence in growth rate and estradiol producing capacity between the F1 or largest (future dominant follicle) and F2 or second largest (future subordinate) growing follicles culminating the process of dominant follicle selection. While the exact mechanisms of dominant follicle selection are not completely understood, there have been many studies on the hormones and factors involved in follicular development. Antral follicles are dependent upon FSH for growth and each follicular wave is preceded by a transient rise in FSH concentrations [4]. Many growth factors linked to regulation of follicular development, such as inhibins, activins, and insulin-like growth factors and their binding proteins, have been identified in follicular fluid of individual bovine follicles [5]. These molecules can regulate follicular cell survival, proliferation, or death. Recent studies have attempted to understand the molecular regulation of follicular development in cattle [6, 7]. However, the molecular mechanisms governing the wave-like pattern of follicular development are incompletely described, particularly at the onset of diameter deviation which is the first morphological indication of follicular dominance.

Traditionally, gene expression studies in the field of follicular development focus on the study of expression of candidate genes of interest. With the development of next-generation sequencing technologies, transcriptome profiling has become a powerful approach for identification of genes globally expressed in various tissues including ovarian follicles [8]. In the present study, we performed RNA-Seq of granulosa cell RNA from bovine ovarian follicles at predeviation (PD) and onset of deviation (OD) stages of a follicular wave in cattle to catalog the transcriptome and identify potential differentially expressed genes associated with these key stages of a follicular wave. This study provides a comprehensive sequence resource for future studies on follicular development in cattle.

## 2. Materials and Methods

### 2.1. Materials

All materials were obtained from Sigma-Aldrich (St. Louis, MO) unless otherwise stated.

### 2.2. Animal Model and Sample Collection

All animal procedures were approved by the Institutional Animal Care and Use Committee at Michigan State University. Estrus was synchronized in nonlactating Holstein dairy cows with two injections of prostaglandin F2*α* (PGF2*α*; Prostamate; IVX Animal Health, St. Joseph, MO) administered 14 days apart, and follicular growth was observed and recorded by daily ultrasonography.

Ovaries were removed from cows at the following stages of the first follicular wave: predeviation (PD; approximately Day 3 after estrus; 1.5 days after emergence [emergence is the first scan where a new follicle at least 4 mm is detected by ovarian ultrasonography]) and onset of deviation (OD; first scan where growth of the F1 [largest; future dominant] follicle to >8.5 mm was detected by ovarian ultrasonography). The F1 follicles were isolated from the PD and OD groups. Granulosa cells were isolated from the two types of follicles (PDF1 and ODF1), lysed, and stored at −80°C immediately.

### 2.3. RNA Isolation

Total RNA was isolated from the lysed granulosa cells using the RNeasy mini kit (Qiagen) and DNase treated on column according to the manufacturer's protocol. The RNA integrity was evaluated by Agilient Bioanalyser and the RNA concentration was measured using a Nanodrop-1000 spectrophotometer. RNA samples with a RNA integrity number greater than 8 were selected for deep sequencing.

### 2.4. Library Preparation and Illumina Sequencing

RNA sequencing was performed by the WM Keck Center for Comparative and Functional Genomics at the University of Illinois at Urbana-Champaign. RNA samples from four follicles were pooled within each group (ODF1 or PDF1). RNA-Seq libraries were prepared with a TruSeq RNA Sample Preparation kit (Illumina) according to the manufacturer's instructions. The cDNA libraries were sequenced on one lane for 100 cycles using Illumina HiSeq*™* 2000 by a TruSeq SBS kit v5 (Illumina) and analyzed with pipeline version 1.8.

### 2.5. Identification of Differentially Expressed Genes and Pathway Analysis

The CLC Genomics Workbench (CLC bio, Aarhus, Denmark) was used to map the sequence reads to the bovine RefSeq database. The reads per kilobase per million reads (RPKM) values were calculated as the normalized transcript expression values [9]. A *Z*-test [10] was used to identify differentially expressed genes between ODF1 and PDF1 (FDR corrected *p* value <0.05, RPKM cut-off > 0.5, and RPKM fold change >1.5) using the CLC genomics workbench. DAVID software (https://david.ncifcrf.gov/gene2gene.jsp) was used to perform GO annotations and KEGG pathway analysis for highly expressed (RPKM > 100) and differentially expressed genes.

## 3. Results

### 3.1. Illumina Sequencing

To identify differentially expressed genes involved in bovine follicular development, Illumina sequencing was used on two libraries constructed from RNA isolated from ODF1 and PDF1 follicles. After filtering, a total of 44,082,301 and 43,708,132 clean reads were obtained from PDF1 and ODF1 libraries, respectively. The clean reads were mapped to the bovine RefSeq database (containing 35,325 annotated transcripts). Using a cut-off value of RPKM > 0.5, a total of 15,533 genes were identified in both types of follicles (Additional file 1: Table S1 in Supplementary Material available online at http://dx.doi.org/10.1155/2016/3472748), among which 719 are considered to be highly expressed (RPKM cut-off > 100) in bovine follicles (Additional file 2: Table S2).

### 3.2. GO Functional Classification and KEGG Pathway Analysis of Highly Expressed Genes

**Figure 1:**
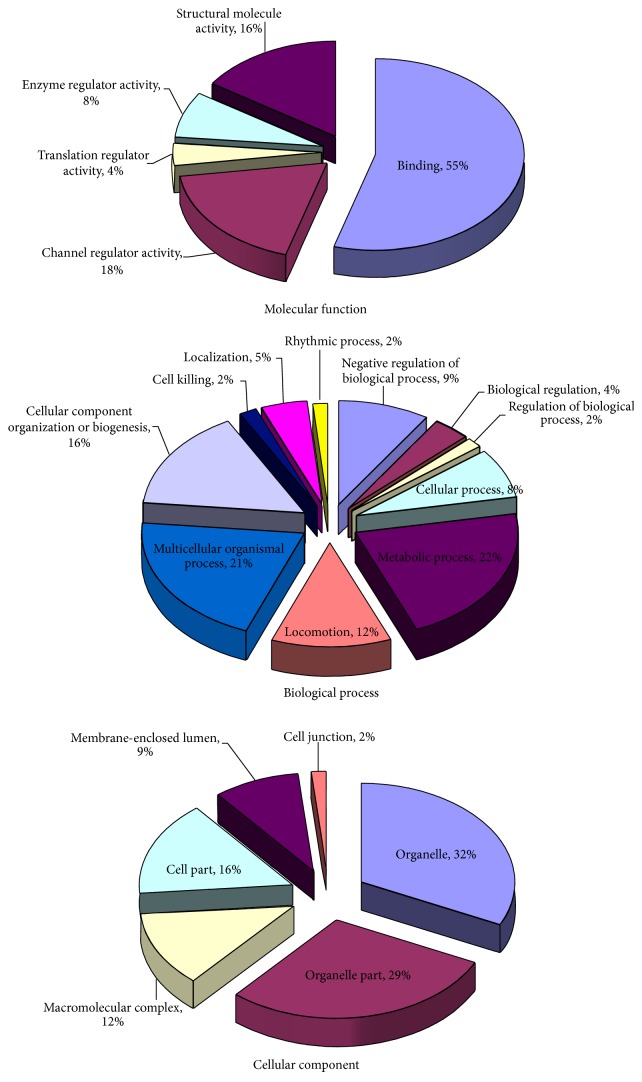
Highly expressed genes GO analysis.

**Figure 2:**
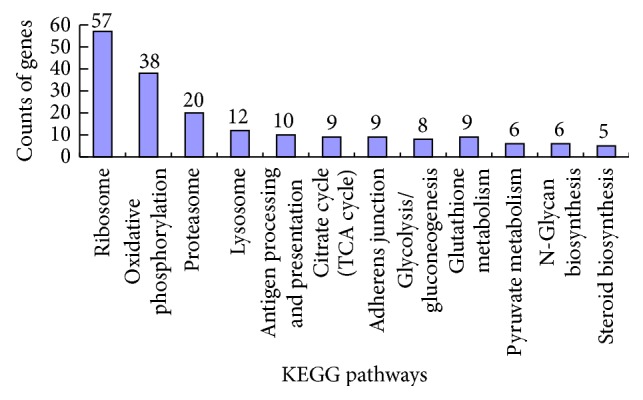
Highly expressed genes KEGG pathways analysis.

The top 30 highly expressed genes in granulosa cells of bovine follicles at the predeviation and onset of deviation stages are shown in Table 1. Many of them are known to be important for follicular growth and development, such as Serpin peptidase inhibitor clade E member 2 (SERPINE2), Inhibin alpha (INHA), Inhibin beta A (INHBA), and Follistatin (FST). GO functional classification of these highly expressed genes was performed using DAVID software. All 719 highly expressed genes can be assigned into 22 groups under three categories (biological process, 39%; cellular component, 44%; and molecular function, 17%) based on their putative functions (Figure 1). Many of the highly expressed genes are involved in metabolic process, multicellular organismal process, and binding. KEGG pathway analysis showed that the highly expressed genes are involved in 12 major pathways (Figure 2), of which the most significantly enriched genes are involved in ribosome pathway.

### 3.3. Differentially Expressed Genes in ODF1 versus PDF1

Using RPKM cut-off > 0.5 and fold change cut-off > 1.5 at FDR corrected *p* value <0.05, a total of 83 differentially expressed genes were identified, with 41 downregulated genes (Table 2) and 42 upregulated genes (Table 3) in ODF1 versus PDF1. To understand the functions of these differentially expressed genes, GO analysis was performed. The upregulated genes were categorized into 14 functional groups under 3 major GO classifications: biological process (35%), cellular component (30%), and molecular function (35%) (Table 4). Many of the differentially expressed genes are known to play a role in ovarian follicular development (Table 5). For example, serine protease 23 (PRSS23) is expressed in granulosa cells and may play a crucial role in follicular atresia, whereas serine protease 35 (PRSS35) is also expressed in granulosa cells and may be involved in ovulation and CL formation and regression. KEGG pathway analysis of the upregulated genes demonstrated that two important genes (CYP11A1 and CYP19A1) in the steroid hormone biosynthesis pathway are upregulated in ODF1 versus PDF1.

## 4. Discussion

Follicular growth occurs in a characteristic wave-like pattern in monotocous species such as cattle [3, 5, 11]. A transient increase in FSH triggers initiation of each follicular wave [5, 11, 12]. Emergence is defined as the first day a new follicle >4 mm in diameter is detected and is the first chronological event marking a new follicular wave that is detectable by ultrasonography. After emergence, follicles in the cohort initially grow at a similar rate (common growth phase) prior to deviation [3]. However, the molecular mechanisms involved regulating the onset of deviation are not well understood, in order to characterize the differences in gene expression that associated with follicular development in different follicles sized in diameter, which the previous studies examined using microarray technology [13–17]. To further investigate the bovine granulosa cell transcriptome and molecular alterations associated with onset of deviation, we examined the transcriptome at specific stages of the estrous cycle.

Illumina sequencing technology was used to determine gene expression levels in ODF1 and PDF1 follicles. A total of 15,533 genes were identified in both types of follicles and 83 of them were identified as differentially expressed between ODF1 and PDF1. Our study provided novel information on the bovine granulosa cell transcriptome and identified specific transcripts highly expressed in granulosa cells of bovine follicles prior to and at onset of deviation, including transcripts encoding for several housekeeping genes (e.g., ribosomal proteins L18a, S27a, and L4) and genes with well-established roles in regulation of ovarian function (e.g., INHBA, INHBB, and FST). Of particular interest was SERPINE2, which is abundantly expressed in granulosa cells of follicles collected at both the predeviation and onset of deviation stages of a follicular wave, illustrating its potential importance in bovine ovarian follicular development. Estradiol and SERPINE2 secretion are positively correlated, but estradiol treatment cannot alter the expression of SERPINE2. FSH and growth factors can directly regulate the expression and secretion of SERPINE2 in granulosa cells, and SERPINE2 is an antiapoptotic factor, which may regulate atresia in bovine follicles [18]. Eleven SERPINE genes are expressed in bovine follicles, but only SERPINE2, SERPINE5, and SERPINE6 are expressed in the granulosa cells [19].

KEGG analysis revealed upregulated genes associated with onset of deviation (CYP11A1 and CYP19A1) involved in the steroid hormone biosynthesis pathways that play an essential role during follicular development. Proteins encoded by CYP11A1 and CYP19A1 genes are members of the cytochrome P450 superfamily, which are monooxygenases that catalyze many reactions involved in steroidogenesis. Previous studies suggested that CYP19A1 was regulated by multiple pathways, including estrogen receptors and cAMP/protein kinase A which are activated by FSHR in granulosa cells, and these regulatory mechanisms are likely critical for acquisition of follicular dominance in cattle [20]. Our transcriptome sequencing data is consistent with these results. The increase in transcript abundance for CYP19A1 in ODF1 versus PDF1 follicles is consistent with the increase in estradiol producing capacity associated with diameter deviation [3].

It is acknowledged that study design was not optimal due to limited biological replication because single pooled samples (*n* = 4 per group) were used in Illumina sequencing analysis. Despite such limitations, results have significantly enhanced understanding of bovine follicle transcriptome composition and potential differences in gene expression associated with follicular development that are foundational to further study in the future; several interesting candidates were revealed for future investigation, particularly genes linked to regulation of cell proliferation and survival. For example, results of present studies suggest that PPM1K, a Mn^2+^/Mg^2+^-dependent protein phosphatase of PPM family, is potentially upregulated with the onset of deviation in the granulosa cell layer of bovine follicles. This protein is critical for cell survival and embryonic development and can regulate the mitochondrial membrane permeability transition pore opening [21]. Potential upregulation of granulosa cell BEX2 and GREB1 transcript abundance was also noted in association with onset of dominance and may be associated with enhanced granulosa cell survival. BEX2 can downregulate apoptosis and activate the JNK (Jun NH2-terminal kinase) pathway, and these effects can be abolished by administration of a JNK specific inhibitor [22]. GREB1 is an estrogen receptor and coactivator linked to cell proliferation and GREB1 expression is estrogen dependent. It is possible that increased expression of PPM1K, BEX2, and GREB1 may be associated with granulosa cell proliferation and survival during the onset of deviation [23]. TNFAIP6/TSG6 is tumor necrosis factor and alpha-induced protein 6; it is suggested that TSG-6 plays a role in cell-cell or cell-cell matrix interactions during inflammation and tumorigenesis. High LH/hCGR gene expression intensity was associated with TNFAIP6/TSG6 gene expression which has a pivotal importance in the mucification of the COC during the preovulatory period [24]. It is suggested that the expression levels of TNFAIP6/TSG6 were nearly 280-fold in granulosa cells of large follicles than that of small follicles [14]. In our study, TNFAIP6/TSG6 was also differentially expressed in ODF1 and PDF1 with a 4.26-fold change. Altogether, these characteristics suggest that TNFAIP6/TSG6 plays a crucial role in accelerating follicle growth during follicular waves in cattle.

## 5. Conclusions

The present study characterized the granulosa cell transcriptome of bovine follicles at specific stages of follicular development and identified 83 differentially expressed genes potentially associated with onset of deviation, many of which are linked to regulation of follicular development. The study provides a foundation for future studies to investigate regulation of granulosa cell expressed genes and the regulatory mechanisms controlling antral follicle development during follicular waves in cattle.

## Supplementary Material

Table S1: Shows all the genes (15,533) expressed in both types of follicles (ODF1 and ODF2) with a cut-off RPKM of 0.5.
Table S2: Shows the highly expressed genes (719) in PDF1 and ODF1 follicles with a cut-off RPKM > 100.

## Figures and Tables

**Table 1:** Top 30 highly expressed genes in ODF1 and PDF1 follicles.

Gene name	PDF1-RPKM	ODF1-RPKM
Glutathione S-transferase alpha 3	12445.32	17611.44
Serpin peptidase inhibitor clade E member 2	6816.847	11075.44
Inhibin alpha	6341.615	9658.832
Inhibin beta A	5824.462	9440.244
Serglycin	8808.27	7315.526
Follistatin	3435.171	4998.088
Cytochrome c oxidase subunit I-like	5017.207	3707.555
Unknown	7026.759	3115.129
Cytochrome c oxidase subunit III-like	3508.943	2440.522
Cytochrome c oxidase subunit I-like	3252.12	2372.119
Unknown	5741.943	2262.251
Milk fat globule-EGF factor 8 protein	2439.639	2077.699
Vimentin	2032.584	2050.732
Lysosomal protein transmembrane 4 beta	1539.453	2025.655
Gap junction protein alpha 1	1408.231	1975.614
Cytochrome P450 family 19 subfamily A polypeptide 1	656.8769	1944.887
Eukaryotic translation elongation factor 1 alpha 1	1864.128	1924.407
Low density lipoprotein receptor-related protein 8 apolipoprotein E receptor	1061.542	1897.644
Enolase 1	1005.447	1707.612
Heat shock protein 8	1615.28	1700.711
Ribosomal protein L18a	1559.355	1680.399
Glyceraldehydes 3 phosphate dehydrogenase	1342.963	1670.066
Ribosomal protein S27a	1576.131	1589.545
Ribosomal protein	1627.751	1566.558
ST3 beta-galactoside alpha-2,3-sialyltransferase 4	1096.954	1542.973
Ribosomal protein L4	1443.707	1502.241
Ribosomal protein S8	1563.222	1490.917
Tribbles homolog 2	1411.494	1461.935
Ribosomal protein S3A	1409.527	1450.511
Cytochrome P450, family 11, subfamily A, polypeptide 1	908.9737	1425.869

**Table 2:** List of downregulated genes in ODF1 versus PDF1 and their functions.

Gene symbol	GenBank number	PDF1 RPKM	ODF1 RPKM	Fold change	FDR corrected *p* value	Gene product functions
ACTR1A	NM_001193248.1	17.31	0.43	−39.45	2.63 × 10^−2^	Vesicle motility
LOC787803	XM_002700116.1	45.66	2.15	−20.79	3.41 × 10^−7^	Unknown
PPP1R14A	XM_002694966.1	68.29	3.99	−16.76	7.75 × 10^−11^	Protein phosphatase inhibitor
OLA1	NM_001046045.1	22.71	1.74	−12.79	1.10 × 10^−2^	Hydrolase activity and GTP binding
QRFPR	NM_001192681.1	19.15	1.55	−12.07	4.40 × 10^−2^	Modulate adenylate cyclase
RMRP	NR_036646.1	149.79	13.57	−10.82		Lncrna class
RN5-8S1	NR_036643.1	9330.16	856.09	−10.68		Unknown
LOC100335749	XR_083021.1	35.26	4.58	−7.54	8.45 × 10^−4^	Senescence-associated protein-like
C11H2orf40	NM_001038113.1	41.02	5.84	−6.88	2.22 × 10^−4^	Esophageal cancer
BOLA	NM_001040532.1	74.93	11.19	−6.56	1.01 × 10^−8^	Transcription
ANGPT2	NM_001098855.1	39.04	7.91	−4.83	3.56 × 10^−3^	Angiogenic signal
VNN1	NM_001024556.2	33.51	7.26	−4.52	1.94 × 10^−2^	Amidohydrolase
KRT2	XM_001254015.1	40.64	9.41	−4.23	6.20 × 10^−3^	Keratinocyte activation
IHH	NM_001076870.2	42.84	9.98	−4.21	3.93 × 10^−3^	Smoothened
RN18S1	NR_036642.1	4431.16	1040.31	−4.17		Unknown
BOLA	NM_001038518.1	101.2	26.48	−3.74	6.16 × 10^−8^	Transcription
LOC100335409	XM_002705970.1	1012.71	275.19	−3.61		Unknown
LOC100140002	XR_084188.1	38.53	10.81	−3.49	3.63 × 10^−2^	Envelope glycoprotein-like
ADM	NM_173888.3	203.61	61	−3.27		Adrenomedullin
4-Sep	NM_001034651.1	69.77	22.91	−2.98	9.40 × 10^−4^	Cytokinesis, platelet secretion
ITPR1	NM_174841.2	61.79	22.18	−2.73	9.78 × 10^−3^	Intracellular channel
RN28S1	NR_036644.1	537.61	205.32	−2.56		Unknown
LOC100336997	XM_002685421.1	5741.94	2262.25	−2.49		Unknown
PRSS35	NM_001035457.3	134.05	55.07	−2.38	1.21 × 10^−5^	Ovulation, CL formation and regression
LOC100337434	XR_083937.1	7026.76	3115.13	−2.21		Unknown
LOC100140226	XM_001787664.2	1183.02	532.17	−2.18		Zinc finger protein 347-like
LOC100337402	XR_083935.1	908.3	413.36	−2.15		Unknown
LOC511901	XM_589328.5	102.29	49.83	−2.01	1.42 × 10^−2^	H1 histone
LOC100137883	XM_002706880.1	98.48	49.64	−1.94	3.47 × 10^−2^	Thymosin beta-4-like
CDH2	NM_001166492.1	103.68	53.3	−1.91	3.44 × 10^−2^	Neuronal recognition
APOA1	NM_174242.3	126.56	66.22	−1.87	1.07 × 10^−2^	Activates spermatozoa motility
PAPSS2	NM_001076075.1	119.75	65.23	−1.8	3.68 × 10^−2^	Skeletogenesis
LOC100299201	XR_084007.1	327.62	178.8	−1.79	1.28 × 10^−7^	Ribosomal protein
GSTA5	NM_001099016.1	138.14	80.02	−1.69	4.93 × 10^−2^	Glutathione transferase
AKR1B1	NM_001012519.1	182.74	108.97	−1.64	1.33 × 10^−2^	Electron carrier activity
SLCO1A2	NM_174654.2	192.47	119.34	−1.58	2.65 × 10^−2^	Mediates transport
HERC1	NM_001103282.1	222.29	138.46	−1.57	1.01 × 10^−2^	Membrane trafficking
CWC25	NM_001105359.1	433.81	274.72	−1.55	7.12 × 10^−6^	Alternatively spliced transcripts
ACOT11	NM_001103275.1	693.81	440.12	−1.54	6.15 × 10^−10^	Acyl-Coa thioesterase activity
LOC615589	NM_001098467.1	245.33	157.07	−1.53	1.10 × 10^−2^	Keratin-like protein
C12H13orf18	NM_001102041.1	269.27	173.55	−1.52	6.34 × 10^−3^	Unknown

**Table 3:** List of upregulated genes in ODF1 versus PDF1 and their functions.

Gene symbol	GenBank number	PDF1 RPKM	ODF1 RPKM	Fold change	FDR corrected *p* value	Gene product functions
GAPDH	XM_001252511.3	1.91	59.65	31.91	9.83 × 10^−11^	Microtubule and NAD binding
BOLA-N	NM_001105651.1	14.28	174.88	12.50		Unknown
PPP1R14A	NM_001193070.1	6.06	65.75	11.08	8.98 × 10^−10^	Smooth muscle contraction
LOC505676	NM_001193296.1	14.04	111.19	8.09		Unknown
LOC100337308	XM_002684003.1	3.72	23.27	6.39	4.40 × 10^−2^	Unknown
MT1A	NM_001040492.2	26.50	150.75	5.81		Bind heavy metals
LOC100125916	NM_001105487.1	14.80	83.88	5.78	1.51 × 10^−9^	Unknown
TNFAIP6	NM_001007813.1	11.17	46.61	4.26	1.06 × 10^−3^	Cell-cell and cell-matrix interactions
BEX2	NM_001077034.1	93.97	345.00	3.75		Mitochondrial apoptosis
GPR85	NM_001075150.2	10.06	35.54	3.61	4.05 × 10^−2^	G-protein coupled receptor
PPM1K	NM_001046474.1	34.75	108.32	3.18	2.52 × 10^−7^	Cellular survival and development
CYP19A1	NM_174305.1	656.88	1944.89	3.02		Estrogen biosynthesis
MT1E	NM_001114857.1	54.11	152.16	2.87	2.61 × 10^−9^	Bind heavy metals
ETNK2	XM_002693881.1	22.35	61.96	2.83	4.65 × 10^−3^	Ethanolamine phosphorylation
CHST11	NM_001192668.1	57.39	154.72	2.75	6.77 × 10^−9^	Biosynthesis chondroitin sulfate
MT2A	NM_001075140.1	49.37	130.49	2.70	4.16 × 10^−7^	Bind heavy metals
PRSS23	NM_001080306.1	58.01	151.79	2.67	2.74 × 10^−8^	Follicular atresia
TXNIP	NM_001101875.2	98.64	231.47	2.40	6.64 × 10^−11^	Oxidative stress mediator
NPR3	NM_174127.2	47.55	106.35	2.28	5.93 × 10^−4^	Natriuretic peptide hormone receptor
GREB1	NM_001205631.1	35.17	78.20	2.27	1.30 × 10^−2^	Estrogen-stimulated cell proliferation
EIF4EBP1	NM_001077893.1	138.11	294.07	2.17	1.30 × 10^−11^	Mediates protein translation regulation
PIK3R1	NM_174575.1	113.42	210.95	1.90	1.21 × 10^−5^	Insulin actions metabolic
LRP8	NM_001097565.1	1061.54	1897.64	1.82		Sperm maturation
SCD5	NM_001076945.1	124.08	210.97	1.74	4.25 × 10^−4^	Energy metabolism
ENO1	NM_174049.2	1005.45	1707.61	1.73		Tumor suppressor
LDHA	NM_174099.2	221.85	366.73	1.69	3.41 × 10^−7^	Affiliated with lncrna
SERPINE2	NM_174669.2	6816.85	11075.44	1.66		Serine protease inhibitor
INHBA	NM_174363.2	5824.46	9440.24	1.65		Regulate gonadal stromal cell proliferation
TMEM176B	NM_001099145.1	106.45	170.39	1.63	2.16 × 10^−2^	Dendritic cells maturation
OAT	NM_001034240.1	395.42	630.73	1.63	1.71 × 10^−11^	Ornithine aminotransferase
CYP11A1	NM_176644.2	908.97	1425.87	1.60		Cholesterol to pregnenolone
OBSL1	XM_002685586.1	221.33	338.40	1.56	1.16 × 10^−4^	Regulate ubiquitin ligase complex
ARFGAP3	NM_001075974.1	273.92	418.22	1.56	5.26 × 10^−6^	Gtpase-activating protein
ITGB5	NM_174679.2	153.24	233.56	1.56	7.68 × 10^−3^	Fibronectin receptor
INHA	NM_174094.3	6341.61	9658.83	1.55		Gonadal hormone secretion
OPTN	NM_001034602.1	160.13	243.40	1.55	5.92 × 10^−3^	Affect cell death
PTGR1	NM_001035281.1	200.07	301.66	1.54	9.40 × 10^−4^	Inactivation of the chemotactic factor
STBD1	XM_002688357.1	143.24	215.66	1.54	2.15 × 10^−2^	Bind to carbohydrates
LOC532189	XR_083049.1	340.18	510.18	1.53	5.69 × 10^−7^	Carboxypeptidase
TMEM20	NM_001076470.1	191.10	286.48	1.53	2.10 × 10^−3^	Solute carrier
ECE1	NM_181009.2	461.24	691.33	1.53	8.98 × 10^−10^	Converts big endothelin-1 to endothelin-1
GNG10	NM_001114512.1	617.73	918.26	1.52	7.59 × 10^−13^	Signal transducer

**Table 4:** GO analysis of genes upregulated in ODF1 versus PDF1.

Category	Term	Genes
Biological process	Hemoglobin biosynthetic process	INHBA, INHA
Biological process	Antigen processing and presentation	LOC505676, LOC100125916, BOLA-N
Biological process	Insulin receptor signaling pathway	EIF4EBP1, PIK3R1
Biological process	Antigen processing and presentation of peptide antigen	LOC100125916, BOLA-N
Biological process	Response to insulin stimulus	EIF4EBP1, PIK3R1
Biological process	Cellular hormone metabolic process	ECE1, CYP11A1
Biological process	Cellular response to hormone stimulus	EIF4EBP1, PIK3R1
Biological process	Regulation of myeloid cell differentiation	INHBA, PIK3R1
Cellular component	Inhibin complex	INHBA, INHA
Cellular component	MHC protein complex	LOC505676, LOC100125916, BOLA-N
Cellular component	Plasma membrane	LOC505676, ARFGAP3, ECE1, GNG10, ITGB5, INHA, LOC100125916, BOLA-N, ENO1
Molecular function	Metal ion binding	ARFGAP3, PTGR1, ECE1, MT1A, CYP11A1, PPM1K, LRP8, SCD5, CYP19A1, ENO1
Molecular function	Peptidase activity	ECE1, SERPINE2, PRSS23, ENO1
Molecular function	Iron ion binding	CYP11A1, SCD5, CYP19A1

**Table 5:** Differentially expressed genes between ODF1 and PDF1 with known or predicted roles in follicular development.

Gene symbol	Fold change	FDR corrected *p* value	Up- or downregulation in ODF1/PDF1	Gene product functions
PPM1K	3.18	2.52 × 10^−7^	Upregulation	Cellular survival and development
BEX2	3.75		Upregulation	Mitochondrial apoptosis
CYP19A1	3.02		Upregulation	Estrogen biosynthesis
PRSS23	2.67	2.74 × 10^−8^	Upregulation	Follicular atresia
GREB1	2.27	1.30 × 10^−2^	Upregulation	Estrogen-stimulated cell proliferation
SERPINE2	1.66		Upregulation	Serine protease inhibitor
INHBA	1.65		Upregulation	Regulate gonadal stromal cell proliferation
CYP11A1	1.60		Upregulation	Cholesterol to pregnenolone
INHA	1.55		Upregulation	Gonadal hormone secretion
TNFAIP6	4.26	1.06 × 10^−3^	Upregulation	Cell-cell and cell-matrix interactions
OPTN	1.55	5.92 × 10^−3^	Upregulation	Affect cell death
PRSS35	−2.38	1.21 × 10^−5^	Downregulation	Ovulation and CL formation and regression
APOA1	−1.87	1.07 × 10^−2^	Downregulation	Activates spermatozoa motility
GSTA5	−1.69	4.93 × 10^−2^	Downregulation	Glutathione transferase
